# Supplementary material for: Plasma Proteome Signature to Predict the Outcome of Breast Cancer Patients Receiving Neoadjuvant Chemotherapy
Source: Cancers (Basel). 2021 Dec 14;13(24):6267. doi: 10.3390/cancers13246267 (PMC8699627; doi:10.3390/cancers13246267)
Supplement: Supplementary file 1 [file cancers-13-06267-s001.zip › cancers-1473468-supplementary/Supplementary Figures.pdf]

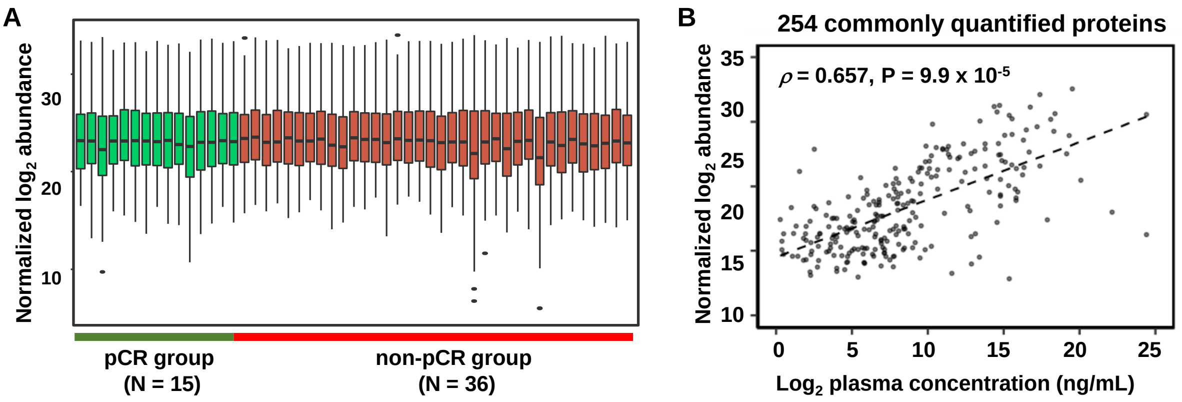

Figure S1. (**A**) Boxplots of normalized plasma protein abundances in the 51 samples (15 BC patients with pathological complete response (pCR) after neoadjuvant therapy and 36 patients with non-pCR) measured by LC-MS analysis. (**B**) Scatter plot of 254 plasma proteins between log2 plasma concentration in the Plasma Proteome Database (bottom) and normalized log2 abundance (Pearson correlation coefficient (ρ): 0.657 and p-value: 9.9×10^{-5}).

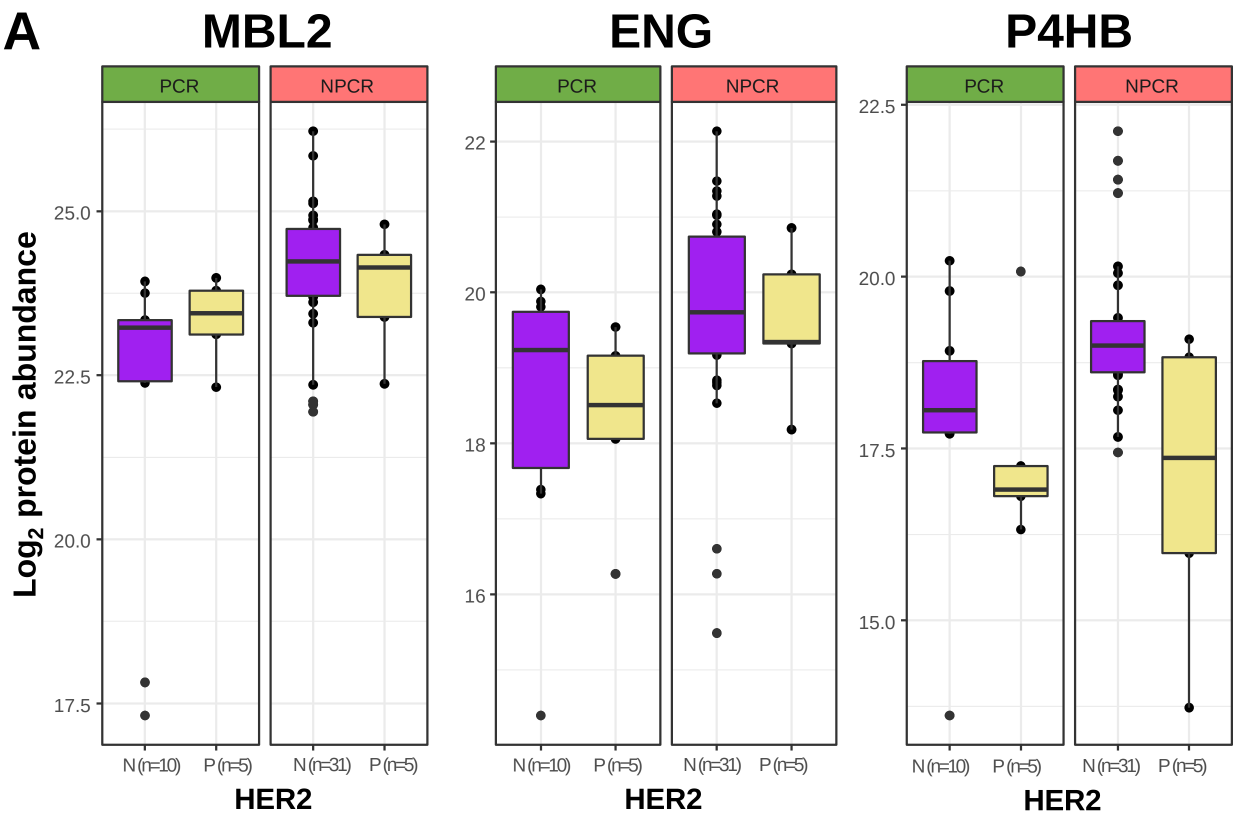

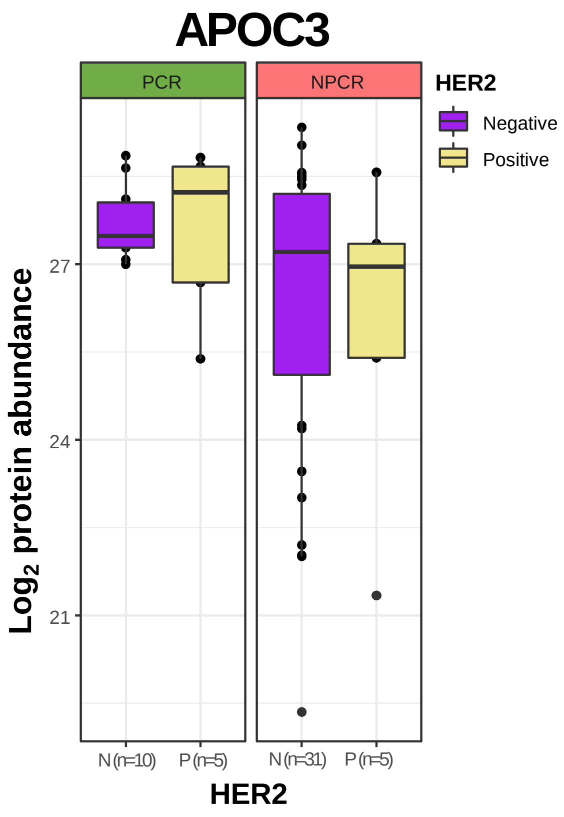

Gene Name	P -value	
	pCR	non-pCR
MBL2	1.56E-01	5.51E-01
ENG	7.76E-01	8.50E-01
P4HB	5.43E-01	8.64E-02
APOC3	9.97E-01	6.18E-01

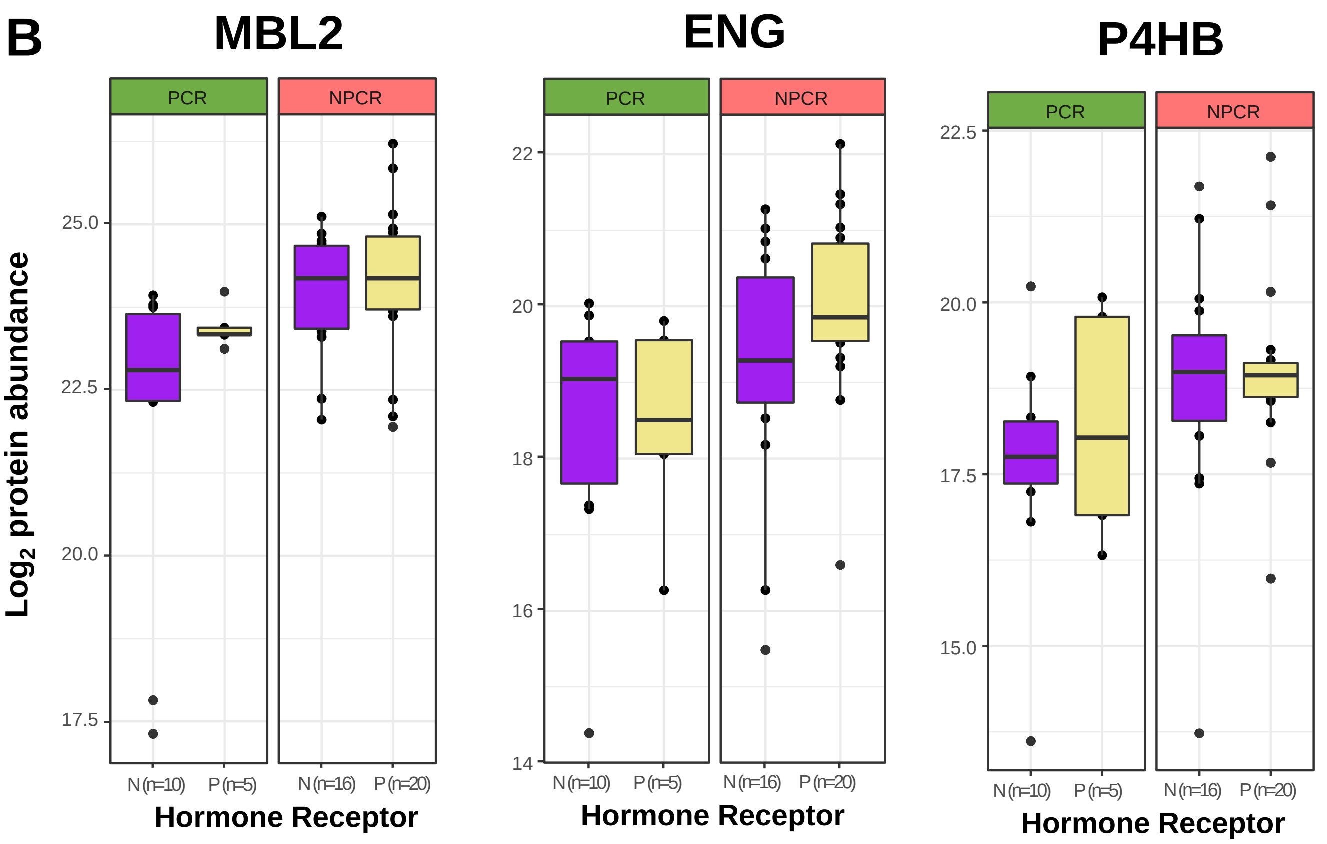

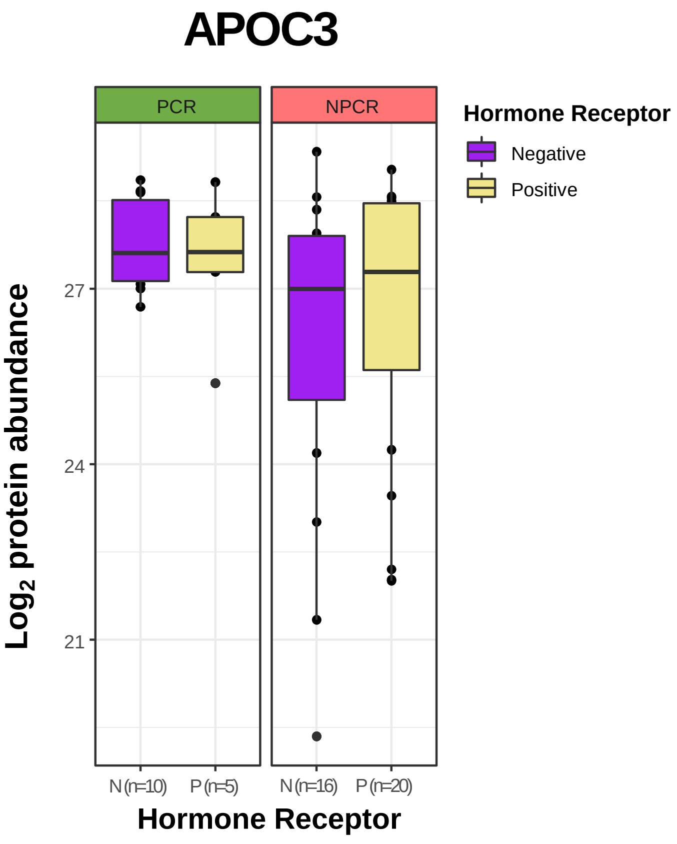

Gene Name	P -value	
	pCR	non-pCR
MBL2	9.97E-02	6.18E-01
ENG	9.69E-01	9.01E-02
P4HB	5.48E-01	6.82E-01
APOC3	6.68E-01	6.46E-01

Figure S2. (A) Boxplot of four proteins (MBL2, ENG, P4HB and APOC3) in groups according to pCR and HER2 presence. (B) Boxplot of four proteins (MBL2, ENG, P4HB and APOC3) in groups divided by pCR and hormone receptor presence.

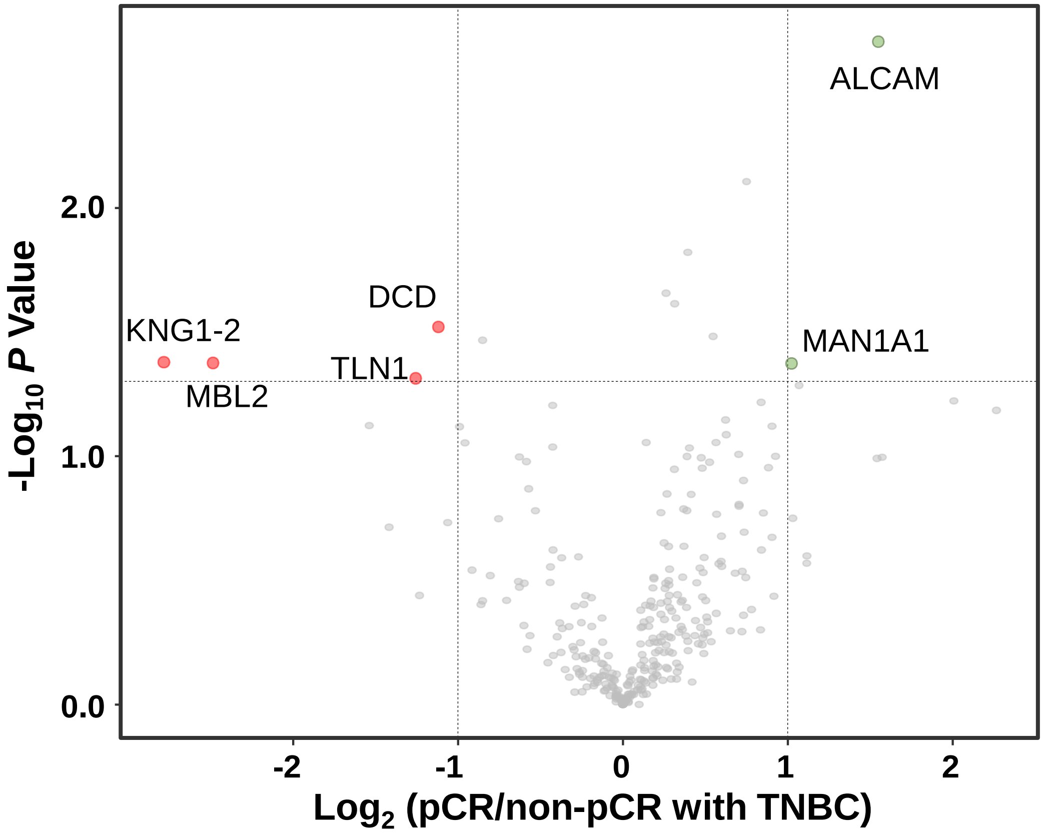

Entry	Gene names	Protein names	Log2 fold-change (pCR/npCR with TNBC)	$-LOG_{10}$(p-value)
P11226	MBL2	Mannose-binding protein C	-2.495	1.386
P01042-2	KNG1-2	Kininogen-1 isoform 2	-2.793	1.389
Q9Y490	TNL1	Talin-1	-1.266	1.324
P81605-2	DCD-2	Dermcidin, isoform 2	-1.128	1.530
Q13740	ALCAM	CD166 antigen	1.538	2.680
P33908	MAN1A1	Mannosyl-oligosaccharide 1,2-alpha-mannosidase IA	1.012	1.384

Figure S3. Volcano plots are depicted with the fold-change of each protein abundance and the *p*-value was calculated by performing a t-test. In the triple-negative BC (TNBC) subtype (n =21), the averages of the plasma proteomic abundance data in the pCR group (n = 8) were compared with the averages of the data for the non-pCR group (n = 13) with TNBC. The red circle shows four plasma proteins showing significant increases in the non-pCR group. The green circle shows two plasma proteins with significant decreases in the non-pCR. Gray circles are plasma proteins with no statistical significance.

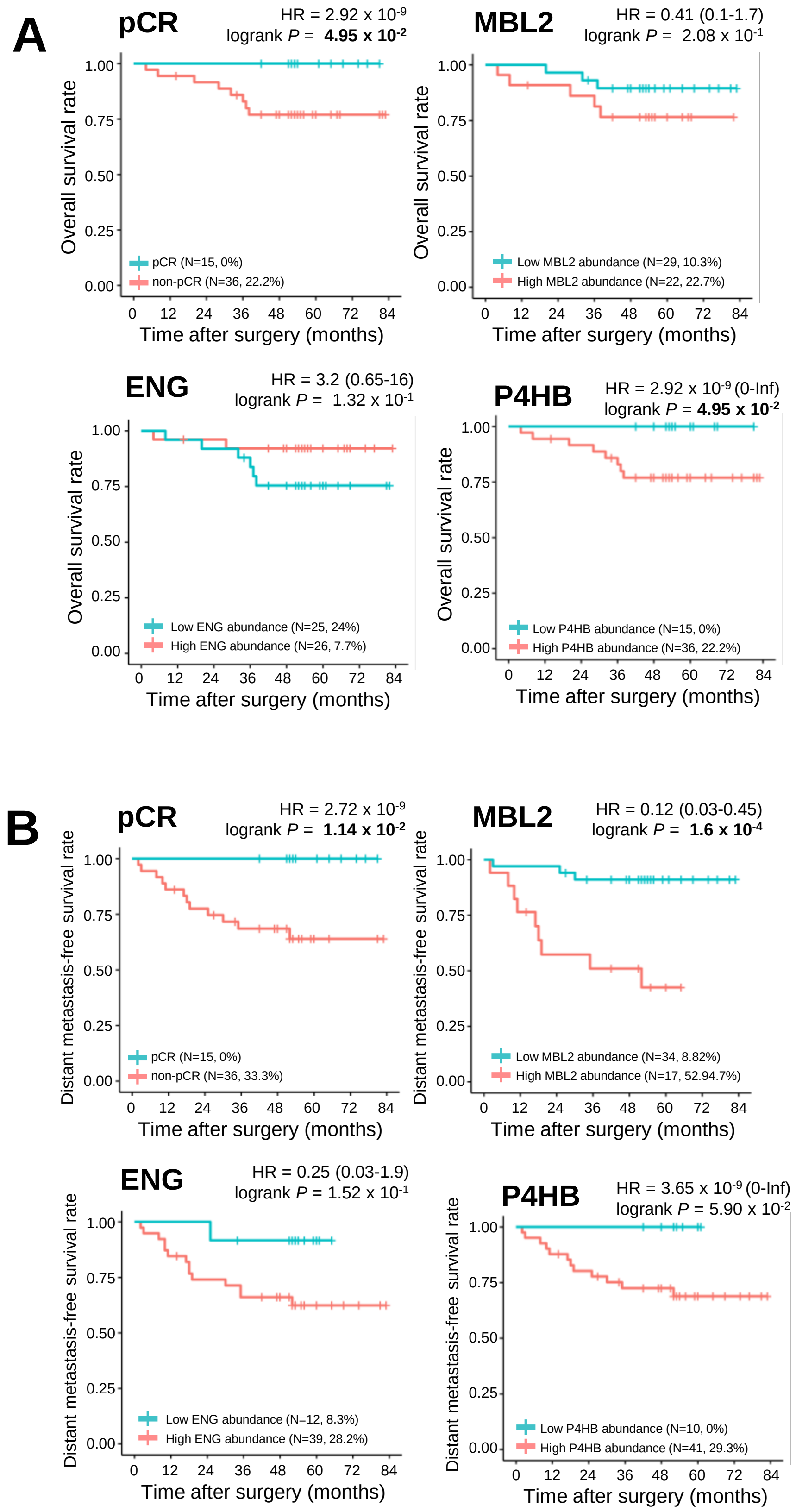

Figure S4. Kaplan-Meier plots of pCR in relation to three proteins (MBL2, ENG, and P4HB) against overall survival (A) and distant metastasis-free survival (B). Statistical significance was determined using the log-rank test. *p*-values≤0.05 are displayed in bold.

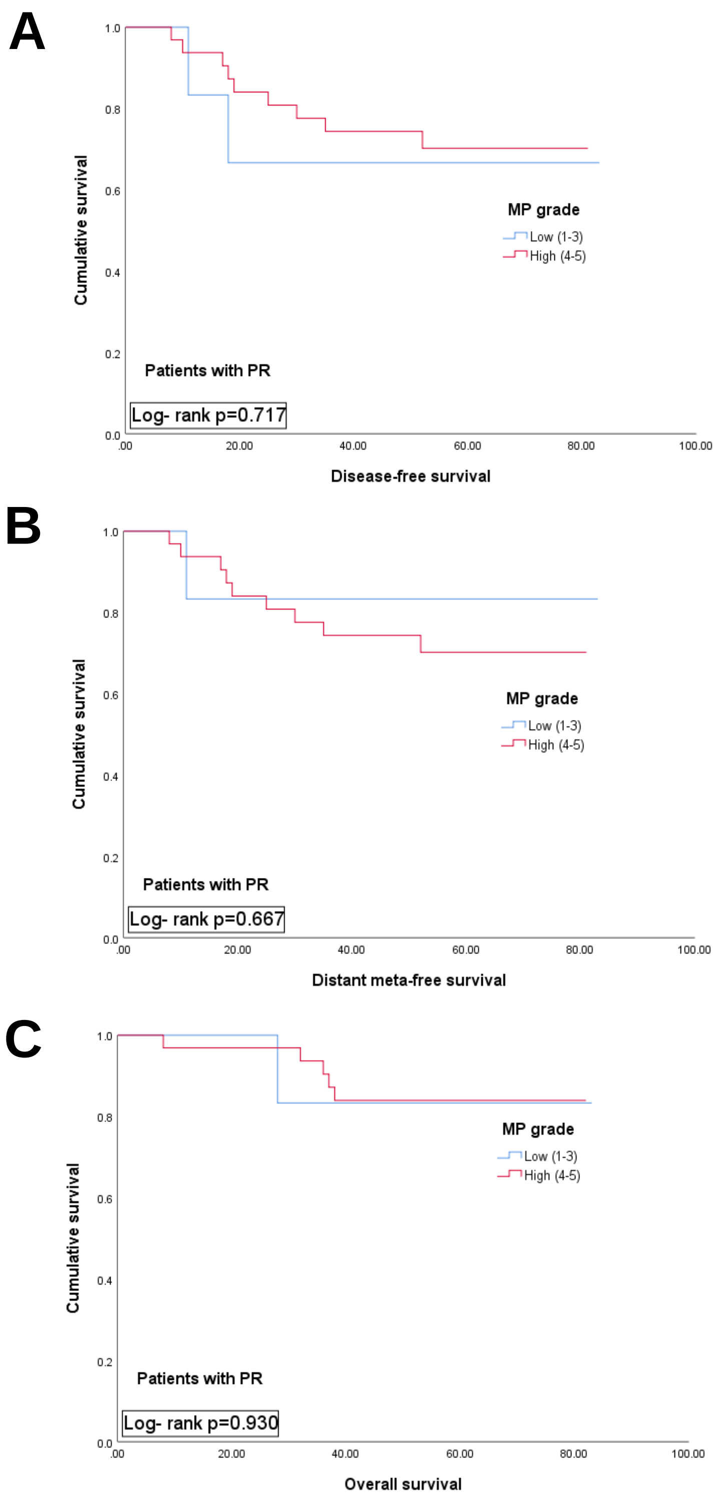

Figure S5. Kaplan-Meier plots of Miller–Payne grades in disease-free survival (A), distant meta-free survival (B), and overall survival (C).
