# Supplementary material for: Plasma Proteome Signature to Predict the Outcome of Breast Cancer Patients Receiving Neoadjuvant Chemotherapy
Source: Cancers (Basel). 2021 Dec 14;13(24):6267. doi: 10.3390/cancers13246267 (PMC8699627; doi:10.3390/cancers13246267)
Supplement: Supplementary file 1 [file cancers-13-06267-s001.zip › cancers-1473468-supplementary/Supplementary Table S6.pdf]

Table S6. Association between three biomarkers with clinical and pathologic stage

**Protein**		**Clinical stage**				***p***
		**0**	**1**	**2**	**3**	
**MBL2**	Low	0 (0%)	0 (0%)	21 (77.8%)	17 (70.8%)	0.570
	High	0 (0%)	0 (0%)	6 (22.2%)	7 (29.2%)	
**ENG**	Low	0 (0%)	0 (0%)	17 (63.0%)	9 (37.5%)	0.069
	High	0 (0%)	0 (0%)	10 (37.0%)	15 (62.5%)	
**P4HB**	Low	0 (0%)	0 (0%)	8 (29.6%)	7 (29.2%)	0.971
	High	0 (0%)	0 (0%)	19 (70.4%)	17 (70.8%)	
**Protein**		**Pathologic stage**				***p***
		**0**	**1**	**2**	**3**	
**MBL2**	Low	14 (53.9%)	3 (11.5%)	6 (23.1%)	3 (11.5%)	0.002
	High	2 (8.0%)	3 (12.0%)	8 (32.0%)	12 (48.0%)	
**ENG**	Low	13 (50.0%)	4 (15.4%)	5 (19.2%)	4 (15.4%)	0.011
	High	3 (12.0%)	2 (8.0%)	9 (36.0%)	11 (44.0%)	
**P4HB**	Low	10 (66.7%)	1 (6.7%)	2 (13.3%)	2 (13.3%)	0.012
	High	6 (16.7%)	5 (13.9%)	12 (33.3%)	13 (36.1%)	
